# Overexpression of native ferritin gene *MusaFer1* enhances iron content and oxidative stress tolerance in transgenic banana plants

**DOI:** 10.1371/journal.pone.0188933

**Published:** 2017-11-30

**Authors:** Karuna Yadav, Prashanti Patel, Ashish Kumar Srivastava, T. R. Ganapathi

**Affiliations:** 1 Plant Cell Culture Technology Section, Nuclear Agriculture and Biotechnology Division, Bhabha Atomic Research Centre, Trombay, Mumbai, India; 2 Plant Stress Physiology and Biotechnology Section, Nuclear Agriculture and Biotechnology Division, Bhabha Atomic Research Centre, Trombay, Mumbai, India; Universidade de Lisboa Instituto Superior de Agronomia, PORTUGAL

## Abstract

Iron is an indispensable element for plant growth and defense and hence it is essential to improve the plant’s ability to accumulate iron. Besides, it is also an important aspect for human health. In view of this, we attempted to increase the iron content in banana cultivar Rasthali using *MusaFer1* as a candidate gene. Initially, the expression of all five genes of the *MusaFer* family (*MusaFer1*-5) was quantified under iron-excess and -deficient conditions. The supplementation of 250 and 350 μM iron enhanced expression of all *MusaFer* genes; however, *MusaFer1* was increased maximally by 2- and 4- fold in leaves and roots respectively. Under iron deficient condition, all five *MusaFer* genes were downregulated, indicating their iron dependent regulation. In *MusaFer1* overexpressing lines, iron content was increased by 2- and 3-fold in leaves and roots respectively, as compared with that of untransformed lines. The increased iron was mainly localized in the epidermal regions of petiole. The analysis of *MusaFer1* promoter indicated that it might control the expression of iron metabolism related genes and also other genes of *MusaFer* family. *MusaFer1* overexpression led to downregulated expression of *MusaFer*3, *MusaFer*4 and *MusaFer*5 in transgenic leaves which might be associated with the plant’s compensatory mechanism in response to iron flux. Other iron metabolism genes like *Ferric reductase* (*FRO*), transporters (*IRT*, *VIT and YSL*) and chelators (*NAS*, *DMAS and NAAT*) were also differentially expressed in transgenic leaf and root, suggesting the multifaceted impact of *MusaFer1* towards iron uptake and organ distribution. Additionally, *MusaFer1* overexpression increased plant tolerance against methyl viologen and excess iron which was quantified in terms of photosynthetic efficiency and malondialdehyde content. Thus, the study not only broadens our understanding about iron metabolism but also highlights *MusaFer1* as a suitable candidate gene for iron fortification in banana.

## Introduction

Micronutrients are indispensable to the structural and functional components of various proteins in biological systems. The protein nature of biocatalysts renders them dependent on coenzymes and cofactors such as iron, zinc, vitamin A and others to carry out reactions which cannot be mediated by the R groups of constituent amino acids themselves [1–2]. Iron is an important micronutrient required for formation of chlorophyll and other redox reactions, hence plants experiencing iron deficiency show interveinal chlorosis in young leaves. This phenotype is observed due to involvement of iron in chlorophyll synthesis [3]. It is accompanied by altered root architecture and molecular response leading to induction of iron transporter and chelator synthesis genes [4]. In human beings, deficiencies of iron can impair cognitive and other physiological functions [5]. Iron deficiency is thus one of the most common and debilitating nutritional deficiencies affecting the vegetarian and vulnerable population in the lower strata of society due to lack of dietary diversification and over-consumption of staples [6] like rice, cassava and banana. In order to circumvent this problem, biofortification of food crops is increasingly being investigated and recently, banana has begun to find favor with micronutrient biofortification programs as it forms a staple food in the tropics. It is rich in potassium and energy [7], but poor in iron content [8]. India is the largest producer of banana having produced 29.7 million metric tons of the fruit in 2013 with an annual world production of 106.7 million metric tons [9]. Although economically important, its production suffers setbacks due to several abiotic stressors which produce oxidative stress and greatly lowers the yield and its nutritional quality. Hence a strategy to achieve both increased iron content and tolerance to oxidative stress is desirable.

Functional characterization of plant ferritins is one of the strategies towards improving the iron status of plants. Overexpression of plant ferritin exemplifies this approach [10]. Plant ferritins are part of the large iron storage protein superfamily, with a capacity to sequester up to 4500 atoms of iron thus protecting the plant from iron induced oxidative stress, as well as buffering the cellular milieu against fluctuations in iron levels. Ferritins have been characterized in several plant species of the legumes, grasses and in the dicot weed *Arabidopsis* [11]. Also, overexpression of pea ferritin in rice imparted tolerance to oxidative stress and biotic stress [12]. Reports by different groups also suggest that ferritin is responsive to different stresses at both transcript and protein levels. Abiotic stressors such as iron overload [13], oxidative stress generated by methyl viologen and photoinhibition [14], induce cellular expression of ferritin. An *Arabidopsis* ferritin mutant was found to be sensitive to both excess iron as well as oxidative stress [15]. Thus, ferritin is a potential candidate for iron enrichment in food crops as evident from the higher iron content obtained in transgenic soybean [16] and rice [17].

The present study describes overexpression of native ferritin (*MusaFer1*) in transgenic banana cv. *Rasthali* which accumulated more iron and displayed improved tolerance to oxidative stress compared with untransformed control plants. Additionally, the transgenic plants showed differential expression of genes from the ferritin family and also the ones related to iron homeostasis thereby pointing to possible changes in systemic flux of iron and hence the observed increase in iron content.

## Materials and methods

### Experimental conditions

Untransformed and transformed i*n-vitro* banana plantlets were regenerated from embryogenic cell suspension cultures (ECS) of banana cultivar *Rasthali* [18].

Banana plantlets grown hydroponically on modified half strength Murashige and Skoog (MS) medium (pH 5.8) [19] were acclimatized for 3 weeks in a growth chamber (Panasonic Healthcare Co. Ltd, Japan) viz., under fluorescence white light (40W) for 16 h light/8h dark photoperiod, 70% RH, 25°C. These plantlets were transferred to modified half strength MS media with 250 μM and 350 μM concentrations of iron and allowed to grow for 10d. Similarly, a different set of plantlets were exposed to half strength MS devoid of iron and supplemented with 300 μM ferrozine for 10d. Plantlets exposed only to modified half MS were taken as experimental controls.

Regeneration of untransformed and transformed *in-vitro* plantlets was done as described later under the section “transformation”. The *in-vitro* plantlets were hardened in pre-autoclaved soil under controlled greenhouse conditions (60–65% RH and 25°C ± 2°C) for a period of three months. Plants were irrigated with tap water every two days.

### Gene expression studies

#### Primer design

Primers used in this study were designed using Primer3Web version4.0.0 (http://bioinfo.ut.ee/primer3/) and their sequences are given in S1 Table. All primers were designed to possess a T_m_ of 56°C for RT-qPCR experiments and a final concentration of 0.8 μM.

#### *In-silico* assessment of *MusaFer1* protein

The *MusaFer1* sequence was analyzed using NCBI database (http://www.ncbi.nlm.nih.gov/) and Banana Genome Hub (http://banana-genome-hub.southgreen.fr/) resources to determine the gene model. ChloroP 1.1 (http://www.cbs.dtu.dk/services/ChloroP/) and SignalP (http://www.cbs.dtu.dk/services/SignalP/) servers were used to determine the presence, length and cleavage site of the signal peptide in the protein. Alignment of *MusaFer1* with other homologs derived from UniProt database (http://www.uniprot.org/blast/) was done to ascertain similarity among the transit peptide (TP) sequences using BoxShade and ExPASy bioinformatics resource portal (http://www.ch.embnet.org/software/BOX_form.html). Further, the sequence was analysed in MEGA6 [20] for phylogenetic analysis with other closely related plant ferritin proteins and promoter analysis was carried out to detect *cis*-elements in the Plant CARE database (http://bioinformatics.psb.ugent.be/webtools/plantcare/html/).

#### Expression profiling of *MusaFer1* under iron stress conditions using reverse transcription-qPCR (RT-qPCR)

Fresh leaf and root samples from plantlets grown hydroponically in high iron (250 and 350 μM) condition and control plantlets grown only in half MS were frozen in liquid nitrogen. These were immediately used for RNA isolation using Concert Plant RNA Reagent (Invitrogen, USA). The lysate was further purified and DNase treated using RNeasy Plant Mini Kit (Qiagen, Germany) which utilizes a column-based method for RNA purification. Sampling included separate pooling of three young leaves and young roots from three independent plantlets to ensure reproducibility [21]. The RNA concentration was checked using NanoDrop 2000 and integrity assessed on a 1.5% agarose gel. First strand cDNA was synthesized using 5 μg of total RNA, Oligo (dT)_18_ primers (Thermo Scientific, USA) and ThermoScript Reverse Transcriptase (Invitrogen, USA) according to manufacturer’s instructions. The cDNA was diluted to 1:20 with molecular grade nuclease free water and stored at -80°C. RT-qPCR was performed using selected primers 47–56 (S1 Table) and SYBR Green Extract-N-Amp PCR ReadyMix (Sigma S4320, USA) on Rotor gene-Q platform (Qiagen, Germany). Briefly, a 12 μL reaction mix consisted of 6 μL (2X) ReadyMix, 2 μL (0.8 μM) of each primer and 4 μL 1:20 diluted cDNA. Adhering to minimal MIQE guidelines, RT -qPCR was carried out using *Musa EF*1α gene as reference gene, as previously reported [21–22]. Also, the absence of DNA and any contamination was verified using a no-RT enzyme control reaction and no-template controls (NTC) respectively together with melt curve analysis. The cycling conditions followed for RT -qPCR were as described previously [21]. Similarly, plants grown hydroponically in iron deficient modified half MS added with 300 μM ferrozine and in modified half MS only were taken as treatment and experimental controls respectively, for deficiency experiments. This was followed by isolation of RNA, cDNA synthesis and RT-qPCR as described above. Data analysis was carried out using MS Excel and statistical analysis was performed on 3 biological replicates for each experiment which was repeated at least twice.

### Gene cloning and banana transformation

#### Amplification of *MusaFer1* cDNA from banana cv. *Rasthali*

*MusaFer1* has an open reading frame (ORF) of 2145 bp with 7 introns and 8 exons and thus RNA was extracted from young banana leaves followed by cDNA preparation as already described. Full length coding DNA sequence (CDS) thus obtained was used to amplify the *MusaFer1* CDS using AFw and ARv primers (S1 Table) flanked on the 5’ends by *Pst*1 and *Kpn*1 sites respectively. The thermal cycling conditions used were: initial denaturation at 94°C for 5 min, 30 cycles of 94°C for 1 min, 63°C for 1 min and 72°C for 2 min and final elongation at 72°C for 10 min. The PCR product was viewed on a 1% agarose gel and a 789 bp band was gel purified using High Pure PCR Product Purification Kit (Roche Applied Science, Germany). The eluted product was cloned in InsTAclone pTZ57R/T (TA) vector for sequence and identity confirmation. The sequence of the cloned *MusaFer1* gene was deposited in GenBank with accession ID KP122962.

#### *MusaFer1* sub-cloning into plant expression vector pCAMBIA1301

The ligated *MusaFer1* CDS from TA vector was digested using *Pst*1 and *Kpn*1 restriction enzymes. The 789 bp band released was electrophoresed and gel purified as described above. This fragment was sub-cloned into the plant expression vector pCAMBIA1301 (CAMBIA, Australia). The resultant plasmid henceforth called pCAMBIA-*MusaFer1* was sequenced [DNA sequencing (MWG, Bangalore)] to confirm the sequence of ligated *MusaFer1* CDS in the resultant construct (S1 Fig). This vector was then electroporated into *Agrobacterium tumefaciens* strain EHA105 using Electroporator 2510 (Eppendorf, Germany) and further used for transformation of banana ECS.

#### Transformation, histochemical assay and generation of transgenic *Rasthali* plants

Banana ECS of cv. *Rasthali* was used as explant for transformation with *A*. *tumefaciens* strain EHA105 harboring pCAMBIA-*MusaFer1* following the method described earlier [18]. The co-cultivated cells aspirated on filter discs in the presence of 3’,5’- Dimethoxy-4’-hydroxyacetophenone [Acetosyringone, (ACS)] were transferred to M2 medium [MS salts added with 2,4-D (1mg/L), biotin (1mg/L), ascorbic acid (10mg/L), malt extract (100mg/L), glutamine (100mg/L), sucrose (4.5%), pH (5.3) and gelrite (0.2%)] + cefotaxime (400mg/L) for three days. Transient overexpression was checked after five days of co-cultivation with *Agrobacterium* harboring the pCAMBIA-*MusaFer1* vector using GUS buffer. Intense blue coloration was observed in transformed ECS compared to the pale yellow colored untransformed ECS (S2A and S2B Fig). This was followed by selection of the transformed cells and embryo formation on banana embryogenic media supplemented with 5 mg/L hygromycin and 400 mg/L cefotaxime for three weeks. Three rounds of selection were carried out every three weeks on the same medium. The transformed ECS on banana embryo induction medium supplemented with hygromycin (5 mg/L) showed pale yellow embryos which subsequently developed into secondary embryos on the same medium (S3A Fig). The embryos were grown on germination media [(MS supplemented with 0.5mg/L 6-Benzylaminopurine (BAP)] for three weeks and then transferred on banana shoot multiplication media (MS supplemented with 2mg/L of BAP). Multiple shoots were obtained on banana germination medium supplemented with 0.5 mg/L of BAP from these embryos (S3B Fig) and later transferred to multiplication medium containing 2 mg/L BAP for obtaining multiple clonal copies (S3C Fig). Well rooted individual plantlets were obtained from the multiple shoots by transfer of excised individual shoots to MS based medium supplemented with 1 mg/L of NAA (S3D Fig) and were later hardened in autoclaved soil under greenhouse conditions (S3E Fig). Histochemical assay for the reporter gene (*uidA*) product was done using small leaf fragments of *in-vitro* plants according to the procedure described earlier [23]. Intense blue GUS staining was seen in the transformed leaf tissue while it was absent in untransformed control leaf (S2C and S2D Fig). Three month old green house plants were used for further molecular analysis.

#### Isolation of genomic DNA and PCR

Five putatively transformed lines selected on hygromycin selection media and showing intense GUS histochemical staining, were subjected to molecular analysis. Total genomic DNA was isolated using young green leaf tissue with GenElute Plant Genomic DNA Miniprep Kit (Sigma-Aldrich, USA) as per the manufacturer’s instructions. PCR was performed using *hygromycin phosphotransferase* (*hpt*II) gene specific primers (S1 Table) present within the T-DNA region of the construct. PCR cycling conditions used were: initial denaturation at 94°C for 5 min, 35 cycles of 94°C for 1 min, 56°C for 1 min and 72°C for 1 min and final elongation at 72°C for 5 min. The PCR product was viewed on a 1% agarose gel in 1X TAE buffer. Genomic DNA from untransformed control plants was used as negative control in these PCR reactions. Amplification of a 788 bp band corresponding to the *hygromycin phosphotransferase* CDS was observed in the transgenic lines, whereas the untransformed control did not show this amplification (S4A Fig).

#### Copy number determination in the transgenic plants by Southern blot analysis

Integration and copy number of the transgene was confirmed by Southern blot using a probe for the *hpt*II gene present within the T-DNA region. Genomic DNA was isolated from young leaves of PCR-confirmed transgenic lines as mentioned above. Genomic DNA (25–30 μg) of transgenic and control plants was digested with *Kpn*1 enzyme at 37°C for 16 hours. The overnight digested DNA was heat inactivated at 80°C for 20 minutes and then purified as already described in the preceding section. Ninety microliters of the purified DNA digest was electrophoresed on a 1% agarose gel made in 1X TAE buffer at 1.5V/cm overnight. The smear on the gel was transferred to a Hybond-N+ nylon membrane (Roche, Mannheim, Germany) through physical blotting overnight. The *hptII* gene was amplified, purified and used for probe preparation using DIG Labelling kit (Roche, Mannheim, Germany). Prehybridization was done at 50°C and hybridization at 45°C, while washing and detection were carried out using DIG High Prime DNA Labelling and Detection Starter Kit II (Roche Applied Science, Germany) as per manufacturer's instructions. As *Kpn*1 cuts only once in T-DNA of *MusaFer1* vector, the number of chemiluminescent bands obtained correspond to the number of integration events in the banana genome. Transgenic lines F1, F3, F17 and F22 showed only a single copy in their genome whereas line F11 showed two copies in its genome (S4B Fig).

#### Estimation of quantum of overexpression in transgenic lines and its effect on other iron metabolism genes

Young leaves of transgenic and control banana cv. Rasthali were used for RNA extraction and cDNA was prepared as described previously. The cDNA was diluted to 1:20 with molecular grade nuclease free water and was used for RT-qPCR. Quantitative RT-PCR was done with primers 5–58 (S1 Table) for an amplicon of approximately 250 bp using SYBR Green Extract-N-Amp PCR Ready Mix (Sigma S4320, USA). *Musa EF1α* gene as reference gene and other genes involved in iron metabolism were used to study the effect of *MusaFer1* overexpression. The cycling conditions were identical to those described in expression profiling of *MusaFer1* under iron stress conditions by RT-qPCR. Absolute expression and expression fold (log2) values were calculated using the delta-delta Ct method [24].

### Physiological parameters

#### Assay for improved tolerance towards oxidative stress

The assay for *in-vitro* tolerance to oxidative stress was carried out using two different stressors, namely 100 μM methyl viologen and 400 μM of iron as FeSO_4_ .7H_2_O. The clonal copies were sub-cultured to obtain shoot culture with relatively uniform dimensions. These along with untransformed control shoots were placed on banana multiplication media added separately with the aforementioned stressors for 7d under 16 h/8 h light dark regime. Malondialdehyde (MDA) content of the cultured shoots exposed to oxidative stress was determined similarly as in [20] as an indicator of lipid peroxidation in stressed conditions. At least three replicates were used in the assay. To assess the tolerance levels of greenhouse hardened *MusaFer1* overexpressing transgenic lines, three month old transgenic and control plants were subjected to methyl viologen treatment [25]. Plants were treated with 15 ml of 100 μM methyl viologen alternately for 10d [26]. After the stress period plants were allowed to recover by normal irrigation with tap water. Untransformed plants were used as experimental controls. A continuous excitation photosynthetic efficiency analyzer (Hansatech Instruments make Model no. Handy-Pea) was used to determine photosynthetic efficiency (F_v_/F_m_) in the leaves of transgenic and untransformed plants after 10d of stress.

#### Estimation of iron and zinc content in transgenic plants

Iron and zinc contents of five selected *MusaFer1* expressing transgenic lines and the control plants maintained in the greenhouse were estimated using ICP-OES (Activa S, Horiba 128 JobinYvon SAS, France). Dried samples from each plant were ground to a fine powder and approximately 200 mg of powder was used for acid digestion in Erlenmeyer flasks as per the modified [27] protocol. 20–25 ml of 5:1 mixture of Nitric acid:Perchloric acid was added and left in the fume hood for 18h at room temperature. The overnight digested samples were then heated using a hot plate at 250°C until white fumes were generated, indicating complete digestion. The samples were cooled, diluted with milliQ water and filtered through Whatman filter paper no. 542 specific for mineral analysis.

#### Histochemical staining

Free hand- cut sections of petiole of three month old plants leaves were vacuum infiltrated in 1:1 freshly made 5% (v/v) HCl and 5% (w/v) K-ferrocyanide (Perls’ stain solution) for 20 min. These were further incubated at room temperature for 30 min followed by washing with distilled water. The sections were observed under light microscope (Eclipse 80i Nikon, Japan). Histochemical studies were done for the petioles, of at least three uniform plants of high expressing line F3 [28].

#### Data analysis

In the present study data was recorded for five transgenic lines with at least three replicates. Iron and Zinc levels in different plant samples were compared using Tukey’s test in one-way ANOVA using Microsoft Office Excel. Assessment of significant difference in gene expression was done by the cut-off criterion, as used previously [29].

## Results

### Bioinformatics analysis of *MusaFer1* sequence

**Fig 1:**
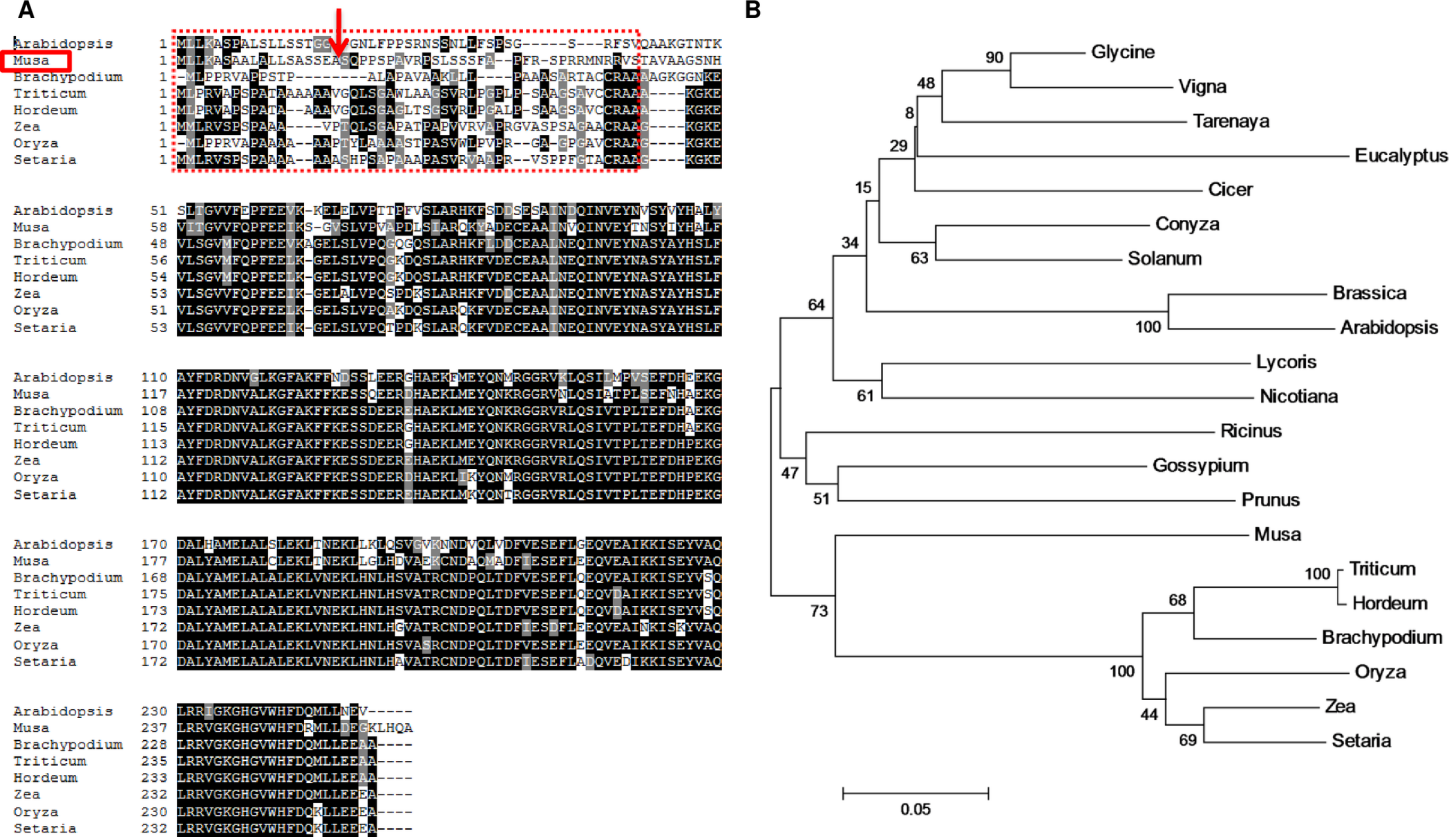
Alignment of *MusaFer1* protein sequence with other homologs. Sequence conservation of *MusaFer1* and non-conservation of the TP when aligned with *Arabidopsis* (D7L9U0), *Brachypodium* (I1IV67), *Triticum* (Q6DQK1), *Hordeum* (B1NC18), *Zea*, *Oryza* (Q8LK80) and *Setaria* (K3ZJM0) were determined. Boxed in red are the N-terminal chloroplast transit peptide sequences of the respective proteins showing non-conservation of the same as opposed to the high degree of conservation in the rest of the protein sequence. The red arrow indicates the cleavage site of *MusaFer1* TP as predicted by SignalP server. UniProt accession numbers are given in parentheses (A). Evolutionary relationship of *MusaFer1* with other ferritin homologs was inferred using the Neighbor-Joining method, bootstrap test (10,000 replicates) and constructed using Clustal Omega and MEGA6 programs (B).

The *MusaFer1* sequence and phylogenetic relationship was elucidated using its protein sequence. It showed 75% homology to the ferritin gene from *Oryza* (Japonica group, Uniprot accession number Q8LK80). Similar to the nomenclature of close homologs, the gene in the present study was named *MusaFer1*. Protein sequence alignment of closely related plant species showed non- conservation of the TP region, whereas the rest of the gene had a high degree of similarity (Fig 1A). A phylogenetic analysis of the protein sequence resulted in formation of two divergent clades, of which *MusaFer1* was closely associated with the monocot clade (Fig 1B). Also, *MusaFer1* sequence analysis in ChloroP showed presence of a TP containing 48 amino acids (S5 Fig) at the N-terminal for localization into the chloroplast. Cleavage of this peptide was predicted between the 18^th^ - 19^th^ amino acids (SEA-SQ) (S6 Fig). Promoter analysis of 5’ upstream region of the transcription start site (TSS) showed presence of the iron dependent regulatory sequence (IDRS) in addition to other light, drought and fungal elicitor responsive *cis-*elements (S7 Fig).

### Physiological and molecular analysis under variable iron concentrations

**Fig 2:**
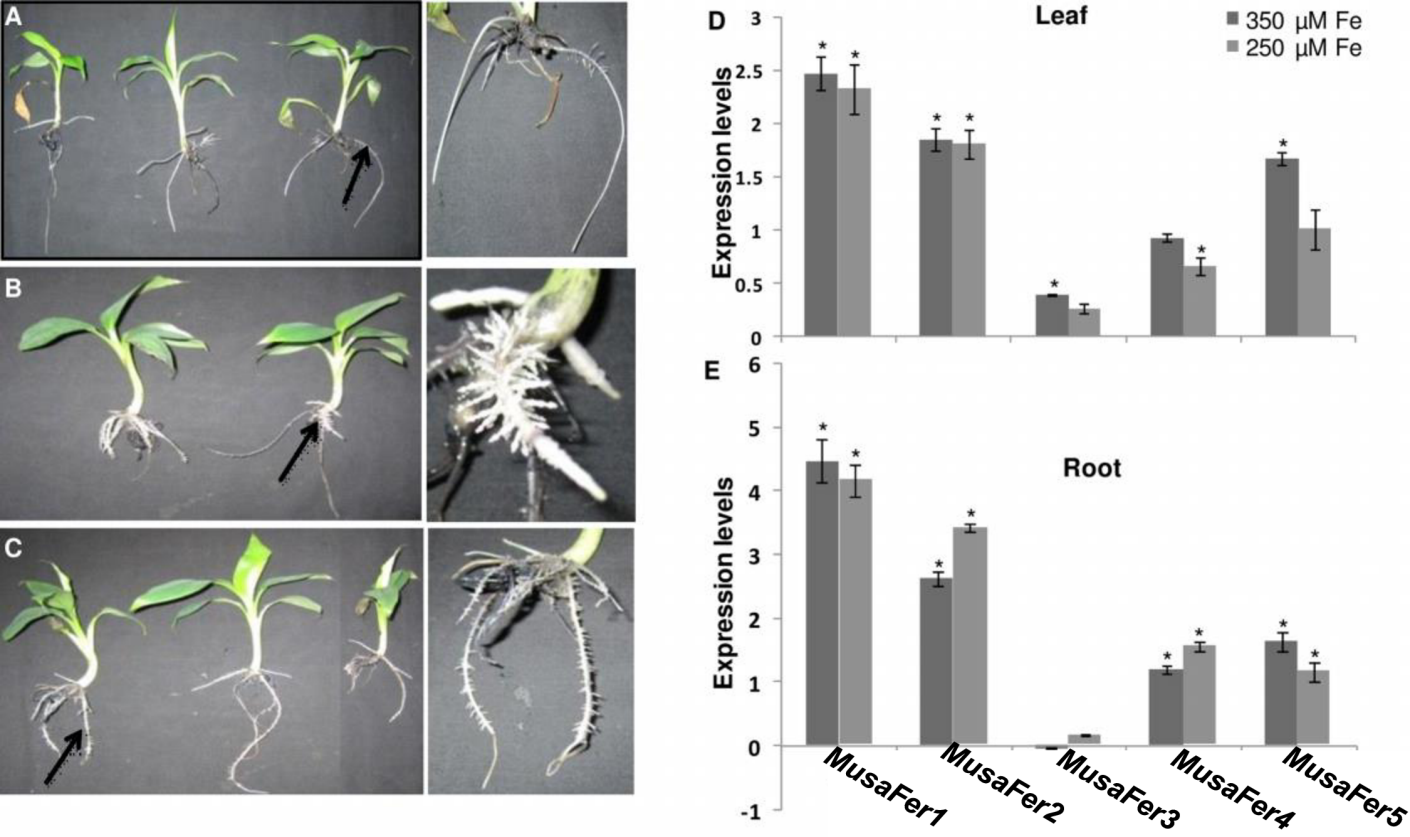
Expression studies under high iron concentrations. Altered root architecture of untransformed control banana plantlets grown in modified half MS with 50 μM iron (A), 250 μM iron (B) and 350 μM iron (C). Under high iron (B and C), roots showed increased lateral branching as indicated by the black arrows enlarged in the insets alongside. Expression levels of *Musa* ferritin genes in 10 day old plants grown hydroponically in modified half MS supplemented with 250 μM iron and 350 μM iron in leaves (D) and roots (E). The values are in triplicates and shown as mean ± SE. *Musa EF1α* is the reference gene used for normalization of *MusaFer1* under different iron conditions. Significance at 0.05 level is indicated by asterisk (*). Expression of *Musa* ferritins in unstressed control plants has been assumed to be 1 for estimating the induction of ferritin in plants grown under excess iron conditions.

**Fig 3:**
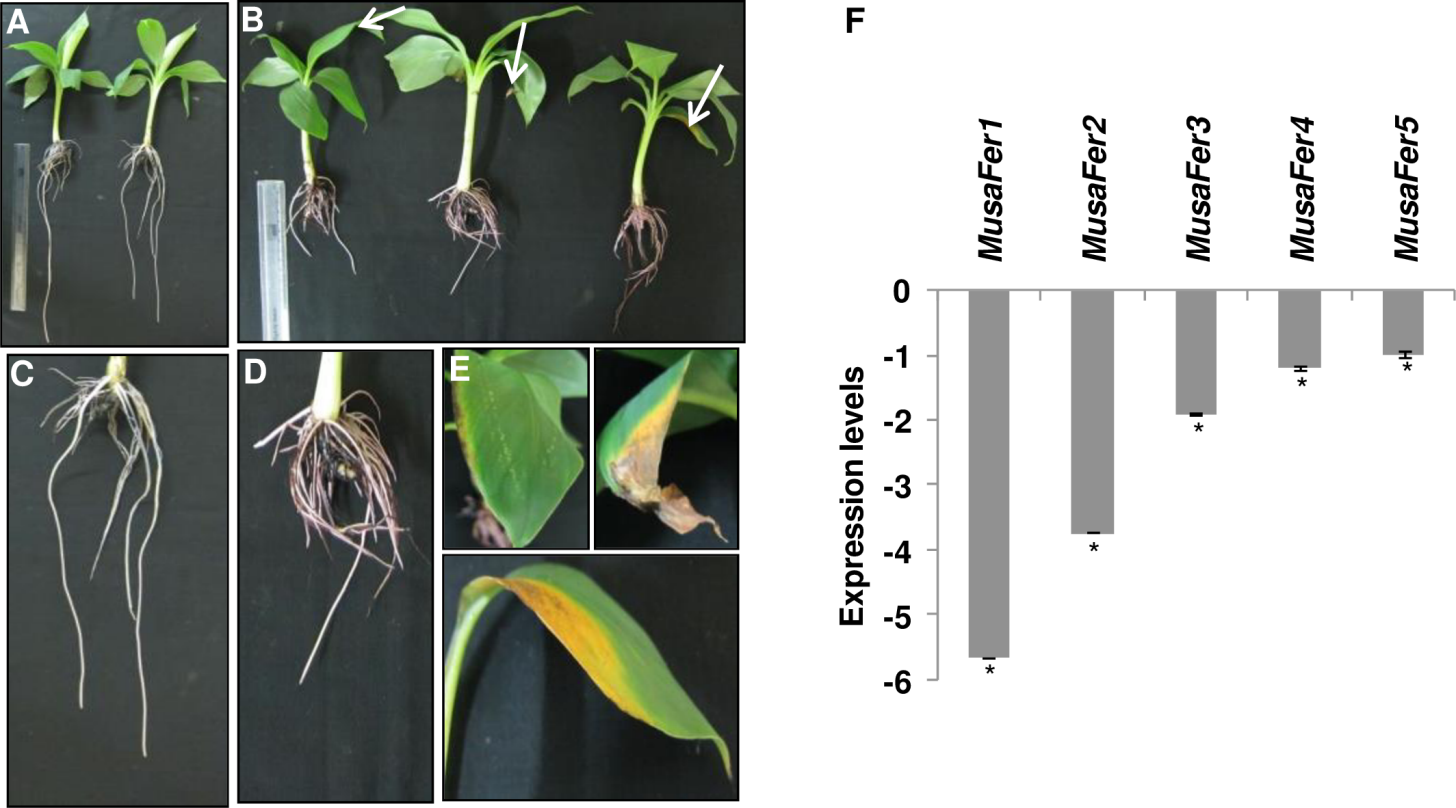
Expression studies under iron deficiency. Untransformed banana plants grown in modified half MS with 50 μM iron (A), 300 μM ferrozine (B). Inset of control roots as in Fig A (C), root of plantlets grown in 300 μM ferrozine as in Fig B (D), inset of leaf margins indicated by white arrows in Fig B (E). Expression levels of *Musa* ferritin genes in leaves of 10 day old plants grown hydroponically in modified half MS without iron and supplemented with 300 μM ferrozine (F). *Musa EF1α* is the reference gene and the values are in triplicates shown as mean ± SE. Significance at p <0.05 level is indicated by asterisk (*). Expression of *Musa* ferritins in unstressed control plants has been assumed to be 1 for estimating the level of ferritin downregulation.

In this study, *in-vitro* plantlets were grown under hydroponics and morphological changes were observed in roots of plant supplemented with increased iron concentrations. On comparison with control (Fig 2A) increased lateral roots emerged throughout the length of the fibrous roots/root cords in presence of 250 μM and 350 μM iron (Fig 2B and 2C). The lower root parts of the plants in 350 μM iron were discolored along with chlorosis near leaf margins and tips on comparison with control plantlets. Also, all the ferritins were found to be induced under excess iron (250 and 350 μM) in both leaves and roots compared to the unstressed control plant. The expressions of *MusaFer1* and *MusaFer*2 were approximately 2-fold up regulated in leaves under both conditions of high iron. Other ferritins namely *MusaFer3*, *MusaFer4* and *MusaFer5* were also upregulated but to a lesser extent except *MusaFer5* which was induced by 1.6 fold under 350 μM iron concentration. In roots, an approximately 4- and 3-fold increase was observed for *MusaFer1* and *MusaFer2* respectively in both 250 μM and 350 μM iron concentrations. Other ferritins were upregulated to a lesser extent except *MusaFer3* which was negligibly downregulated in 350 μM iron. Induction of *MusaFer1* was highest among all other ferritins in both leaves and roots under both iron excess conditions (Fig 2D and 2E). Contrastingly, under iron deficiency, control plant roots were longer [approximately 33 cm (Fig 3A and 3C)] when compared with the stunted root cords (approximately 10 cm) of plants in iron deficient medium (Fig 3B and 3D). Also, iron deficiency manifested as chlorotic leaf tips and margins (Fig 3E) and all the ferritins were downregulated to different levels. *MusaFer1*, *MusaFer2*, *MusaFer3*, *MusaFer4 and MusaFer5* were downregulated by approximately 5.7, 3.7, 1.9, 1.2 and 0.98 folds respectively (Fig 3F).

### Estimation of quantum of overexpression in transgenic lines

**Fig 4:**
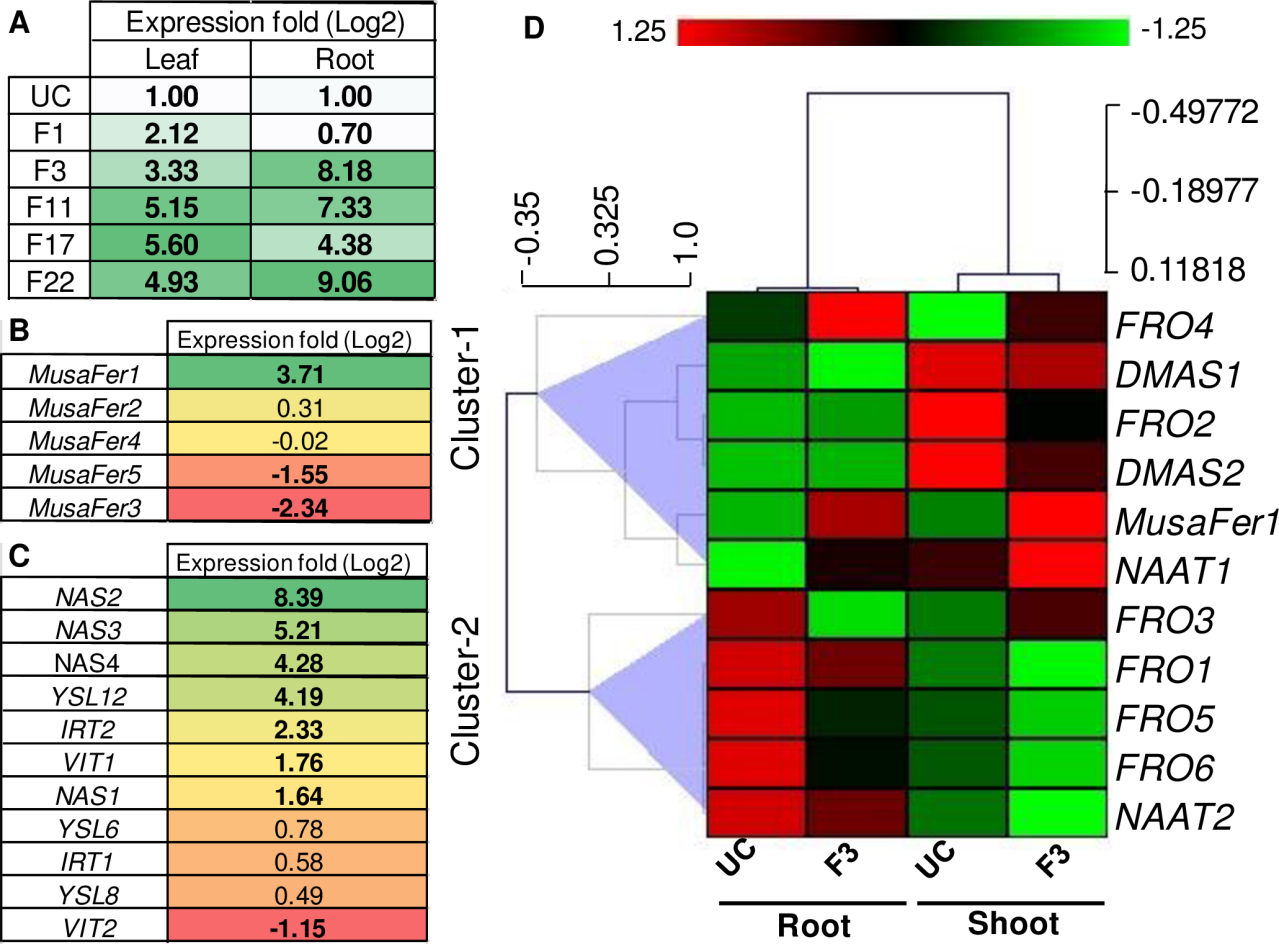
Expression analysis of *MusaFer1* in transgenic lines and effect of its overexpression on other native genes. Expression levels of *MusaFer1* in leaves and roots of transgenic lines (A). Effect of overexpression of *MusaFer1* in the F3 line on other *Musa* ferritins: *MusaFer2*, *MusaFer3*, *MusaFer4* and *MusaFer5* in leaves (B), chelator (*NAS*) and transporters: *YSL*, *VIT* and *IRT* genes in root (C), *Ferric reductase* (*FRO*), *Deoxymugenic acid synthase* (*DMAS*) and *Nicotianamine aminotransferase* (*NAAT*) genes in leaf and root in the form of a heat map (D). The heat-map represents the average differential change in transcript levels from two biological replicates. The data was clustered into two distinct clusters. Cluster-A represents relative upregulation or unchanged transcript levels whereas cluster-B represents the relative down regulation of transcripts in both root and leaves. The ranking analysis was performed using differential expression of the above mentioned genes in leaves and root. The absolute expression change can be found as S2 Table, S3 Table, S4 Table and S5 Table.

To estimate *MusaFer1* overexpression relative to *MusaFer1* in untransformed controls where the expression level is taken to be one [21], RT-qPCR was carried out using *MusaEF*1α as reference gene. A 2.12 fold increase was seen in line F1 whereas 3.33, 5.15, 5.6, 4.93 fold increase was seen in F3, F11, F17, F22 lines respectively in the leaf tissue. Expression levels in roots were 0.7, 8.18, 7.33, 4.38 and 9.06 fold in the transgenic lines F1, F3, F11, F17 and F22 respectively (Fig 4A). On the basis of GUS histochemical staining, single transgene copy number and extent of upregulation, transgenic line F3 was used for further RT-qPCR experiments to assess the effect of overexpression of *MusaFer1* on other genes involved in iron homeostasis.

### Effect of *MusaFer1* overexpression on iron homeostasis genes

Reverse transcription-qPCR analysis was done to assess the effect of overexpression of *MusaFer1* on iron homeostasis genes. Among the five ferritin genes currently annotated in the banana genome, *MusaFer1* overexpression led to down regulation of *MusaFer3* by 2.34 fold and *MusaFer*5 by 1.55 fold in leaves whereas *MusaFer2* was marginally up regulated by 0.31 and *MusaFer4* was down regulated by -0.02 fold (Fig 4B). Among the transporter and chelator genes, *Yellow Stripe-Like* (*YSL*) and *Nicotianamine Aminotransferase* (*NAS*) from annotated banana genome were found up regulated to varying degrees in roots. The *NAS1-4* genes of *Musa* were up regulated in the range of 1.64–8.39 fold (Fig 4C) whereas the *YSL*s were up regulated by 0.49–4.19 fold. In the roots, *Vacuolar Iron Transporter* (*VIT*); *VIT1* was up regulated by 1.76 and *VIT*2 was down regulated by -1.15, while *Iron Regulated Transporter* (*IRT*); *IRT1* and *IRT2* were up regulated by 0.58 and 2.33 folds respectively (Fig 4C). In the leaves of F3 both *FRO1* and *FRO3* were up regulated by 2.01 fold while *FRO4*, *FRO5* and *FRO6* were upregulated by 4.04, 1.04 and 0.89 folds respectively, *FRO2* was downregulated by -1.01 fold. Among the two *Deoxymugeneic acid synthase* (*DMAS*) genes; *DMAS1* was negligibly affected with 0.07 fold upregulation while *DMAS2* was downregulated by -0.55 fold. The two *Nicotianamine aminotransferase* (*NAAT*) genes were reciprocally regulated showing upregulation of *NAAT1* by 0.72 fold while downregulation of *NAAT2* by -0.77 fold. In roots, except *FRO2* and *FRO4* which were upregulated by 0.82 and 0.8 folds respectively, other *ferric chelate reductase* genes *FRO1*, *FRO3*, *FRO5* and *FRO6* were down regulated by -1.53, -3.6, -3.76 and -3.13 folds respectively. Expression of *NAAT* genes in roots showed similar trend to its expression in leaves with *NAAT1* upregulated by 1.2 fold and *NAAT2* downregulated by -1.47 fold while in roots *DMAS1* was downregulated by -0.97 fold and *DMAS2* was upregulated by 2.25 fold (Fig 4D).

### Stress bioassay on *MusaFer1* overexpressing lines

**Fig 5:**
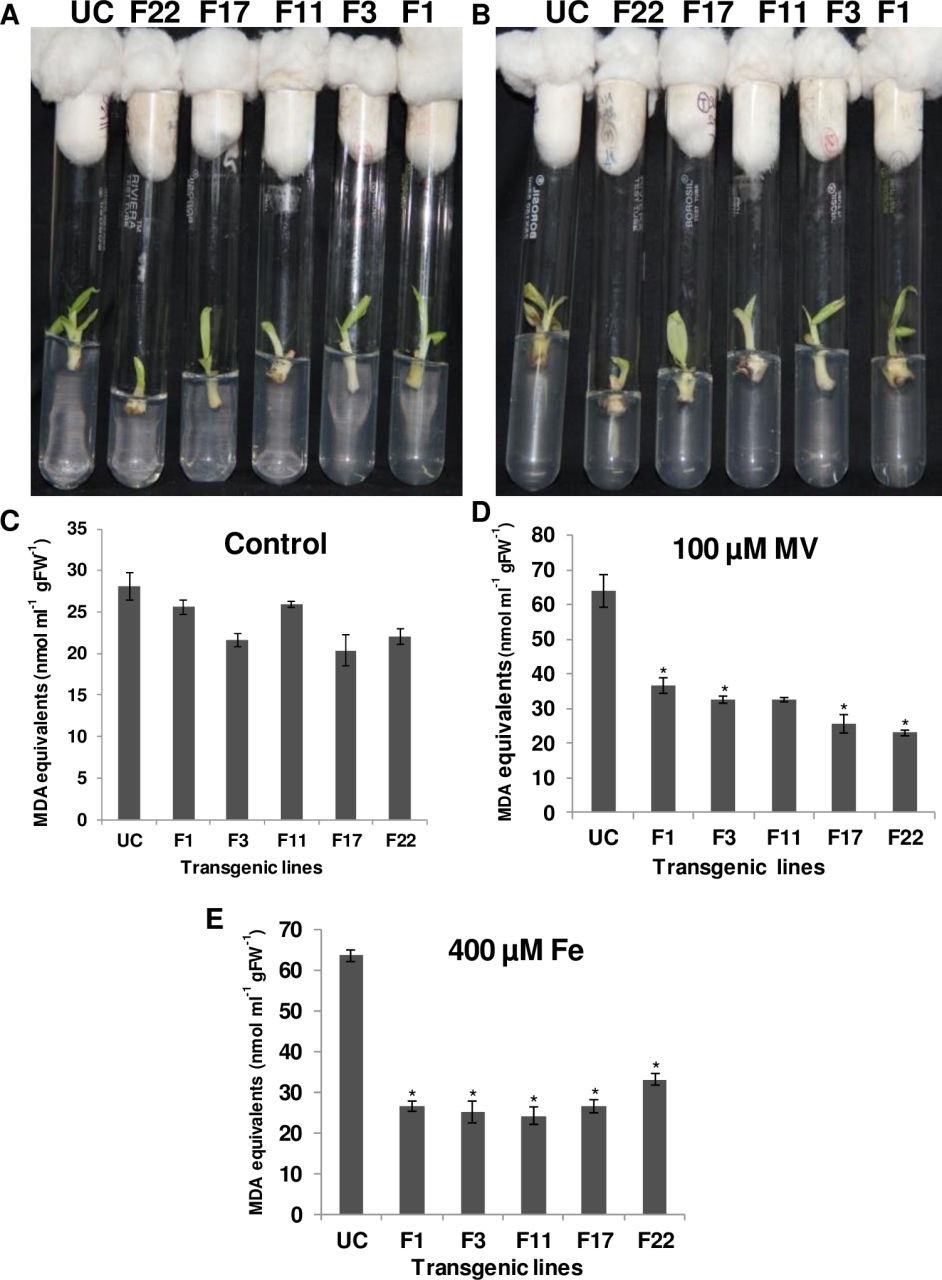
*In-vitro* stress tolerance assay of *MusaFer1* transgenic lines. *In-vitro* transgenic and control shoots subcultured on banana multiplication medium added with 100 μM methyl viologen at day 0 of treatment (A), after day 7 of treatment (B), MDA equivalents under control conditions before stress treatment (C), under 100 μM methyl viologen (MV) stress (D) and 400 μM iron stress (E) after 7d. The bar represents mean of three samples ± SE and asterisk (*) shows significance at 0.05 level compared to the untransformed control.

**Fig 6:**
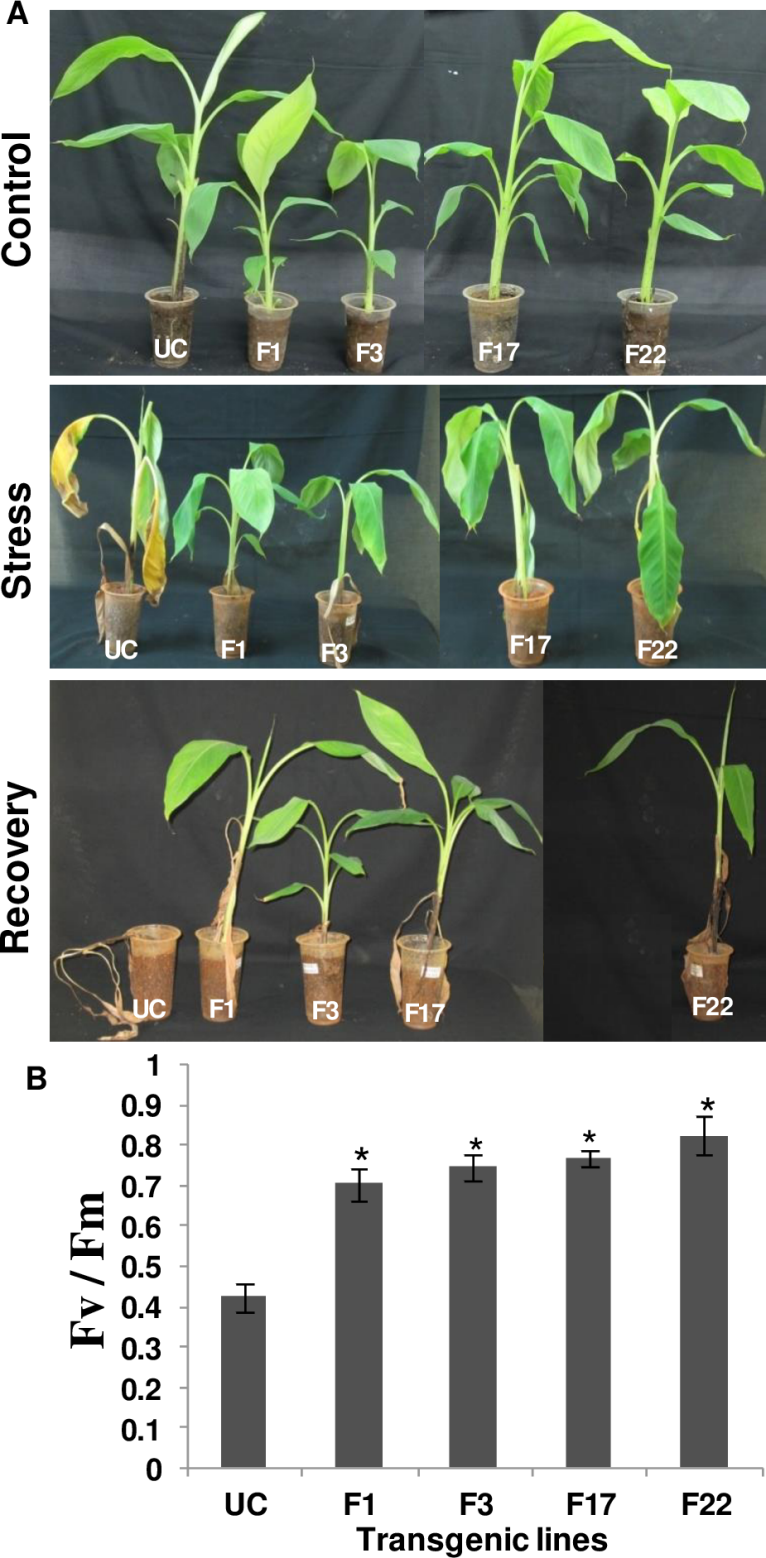
Methyl viologen stress assay. Three month old plants hardened in green house at day 0 of treatment i.e. control condition, after 7d of methyl viologen (100 μM) stress showedwilting and drooping of leaves and recovery after 2 months of irrigating the plants with normal tap water (A). The control plant failed to recover whereas the transgenic lines were resistant to the stress. Photosynthetic efficiency (F_v_/F_m_ ratio) of untransformed (UC) and transgenic leaves (F1, F3, F17 and F22) exposed to methyl viologen (B). The bar represents mean of three samples ± SE and asterisk (*) shows significance at 0.05 level compared to the untransformed control.

Under *in-vitro* methyl viologen stress, the untransformed control showed browning on comparison with transgenic lines subjected to 100 μM methyl viologen (Fig 5A and 5B). MDA levels were decreased by 43% (F1), 49% (F3 and F11), 60% (F17) and 64% (F22) compared with the untransformed control (Fig 5C and 5D). Under 400 μM iron, phenotypically no significant difference was observed between transgenic lines and untransformed controls even after 7d of stress. However, MDA levels were determined to ascertain the effect (if any) of this stress on cellular membranes. MDA levels were decreased by 58% (F1), 60% (F3), 62% (F11), 58% (F17) and 48% (F22) compared to the untransformed control (Fig 5C and 5E). Under greenhouse conditions, leaves of the equivalent untransformed control plant became chlorotic and wilted when compared with the transgenic plant after 10d of methyl viologen stress. The transgenic plants recovered from the stress and continued to grow after two months of the initial stress under routine irrigation in the greenhouse whereas the untransformed control plant succumbed to the oxidative stress induced by methyl viologen (Fig 6A). Photosynthetic efficiency was quantified in terms of F_v_/F_m_ ratio in the leaves of transgenic and untransformed plants. The F_v_/F_m_ ratio for the transgenic lines ranged between 0.64–0.82 whereas the untransformed control showed a mean value of 0.42 (Fig 6B).

### Iron and zinc estimation and Perls’ staining of transgenic plants

**Fig 7:**
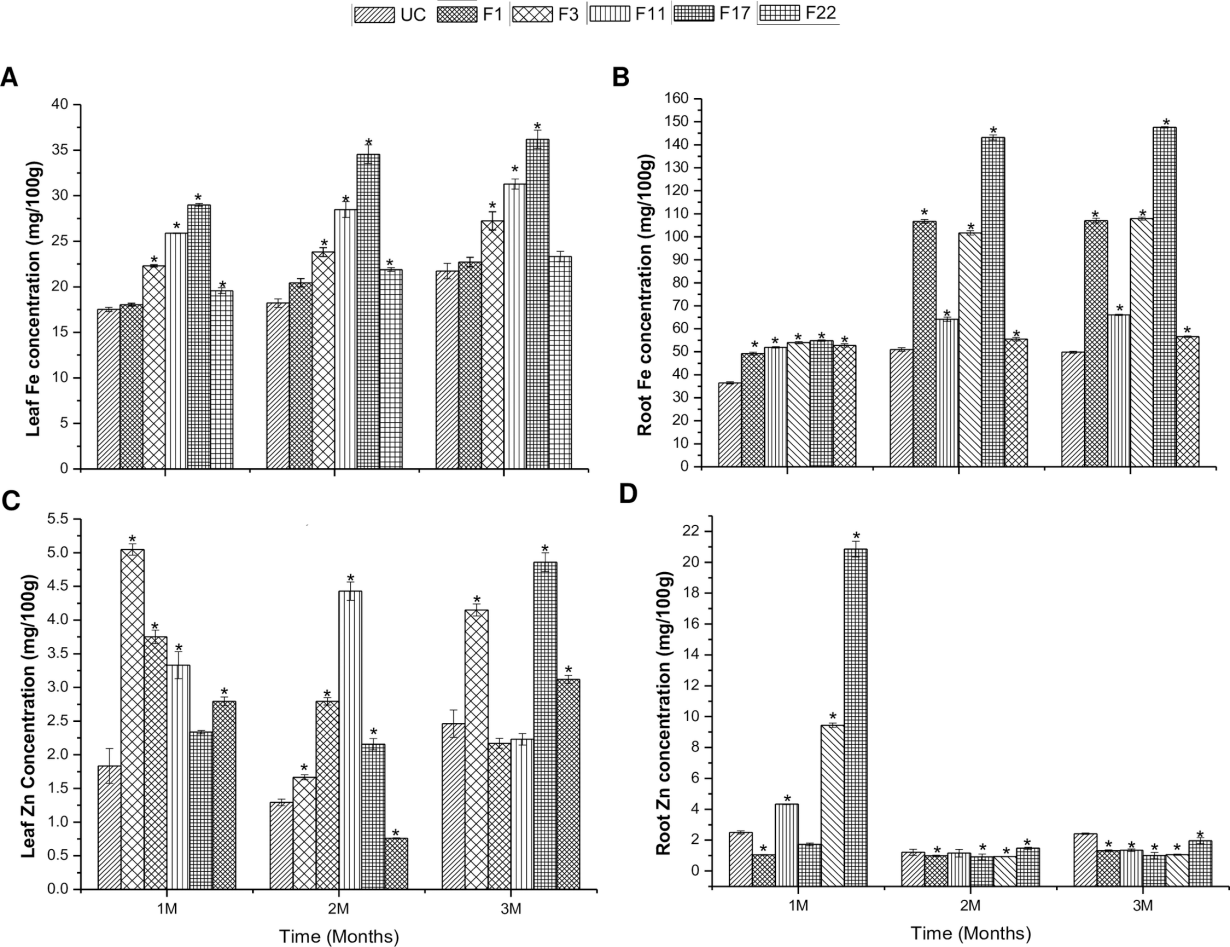
Iron and zinc estimation in *MusaFer1* lines. Iron and zinc estimation in leaves (A, C) and roots (B, D) of transgenic and untransformed control plants. Each bar represents mean of three samples ± SE. Asterisk (*) shows significance at 0.05 level compared to the untransformed control.

**Fig 8:**
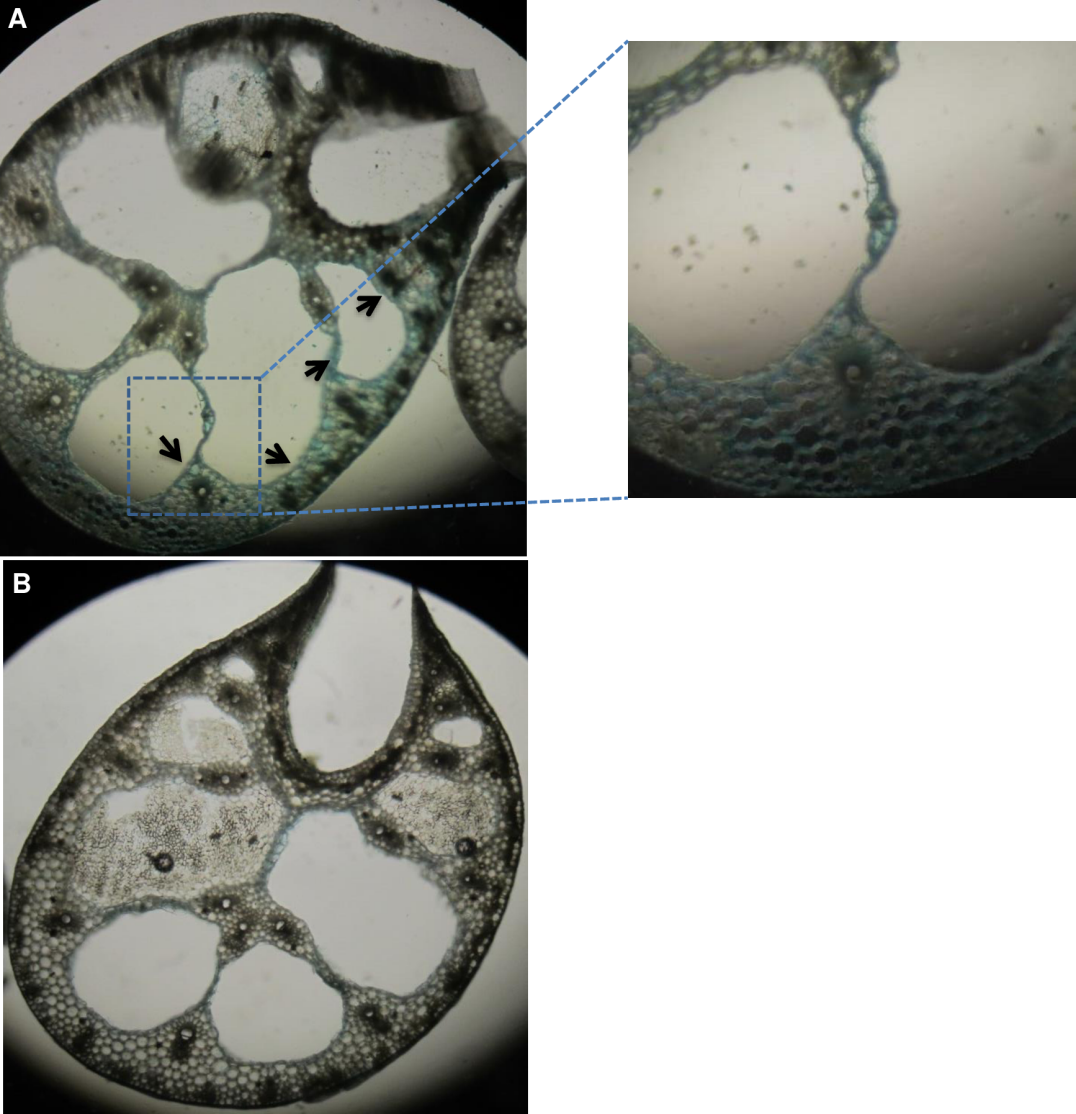
Perls’ histochemical staining. Perls’ staining of petiole of transgenic plant F3 overexpressing *MusaFer1* with an inset alongside of the dotted square boxed area (A) and untransformed plant petiole (B).

Iron and zinc levels were measured in the transgenic leaves and roots by ICP-OES at the age of 1–3 months. Roots of all one month old transgenic lines showed significant increase in the iron content compared to control, while uptake in leaf was significant for all except in line F1. The same trend was observed after two and three months in both leaves and roots. In leaves, the range of iron varied from 18–36 mg/100g across three months (Fig 7A) while it was 49–148 mg/100g in roots (Fig 7B). Histochemically, iron deposition was observed through Perl’s staining of F3 leaf petiole. It showed higher deposition of iron on comparison with the petiole of untransformed control (Fig 8). A significant increase of zinc content was observed in the leaves of all the transgenic plants at two months, whereas transgenic line F17 did not show significant uptake at one month. Lines F3 and F11 did not show significant uptake at three months compared to untransformed control (Fig 7C). The zinc concentration in roots was significant in all transgenic lines after one, two and three months but were not consistent between transgenic and control plants (Fig 7D). The iron and zinc concentrations were thus higher than the respective controls at all time point in leaves and roots but were not consistent with each other.

## Discussion

The iron status of crop plants has a significant impact on human health, necessitating measures for nutritional improvement. Towards this goal, transgenic technology has been explored for development of biofortified crops enriched in iron [30]. Overexpression of candidate genes such as ferritin has yielded a direct positive correlation with the iron status of many food crops. Among these, soybean ferritin has been extensively overexpressed in the past and has been shown to improve iron content in the transgenic plants [10–12, 25]. In the present study, on the basis of induction levels, one native *MusaFer1* was overexpressed in banana cv. Rasthali to determine the potential of this native gene in increasing the iron content in banana leaves and roots.

The *MusaFer1* sequence was predicted to contain a 48 amino acid long N-terminal chloroplast TP whose cleavage was predicted between 18^th^-19^th^ amino acids (SEA-SQ). Other studies report the presence of a 51 amino acid—long TP in higher plants. However, as depicted in the alignment of different plant ferritins, these signal peptides range from 36–48 amino acids and do not show significant similarity as previously reported [31]. Chloroplast transit peptides are present in nuclear-encoded plastidic proteins, signaling them for import into the chloroplast. Although not fully conserved, the TP probably has some common structural domain thereby targeting the ferritins to the chloroplast. Furthermore, *in silico* 5’ upstream sequence analysis showed a number of other *cis*-acting elements in addition to IDRS as mentioned earlier, which are responsive to methyl jasmonate, Myb/drought and light. Stresses such as drought, cold, salinity, high light and pathogen attack converge upon production of ROS [32] which in turn induces ferritin synthesis [33]. In plant defense responses to wounding and biotic stress, the resultant signaling by methyl jasmonate uses nitric oxide as a mediator [34–35]. Interestingly, NO is essential for ferritin induction in oxidative stress, such as that caused by Fenton reaction under excess iron conditions [13], pointing to hormonal regulation of iron homeostasis and oxidative stress. Ethylene, jasmonic acid, auxin metabolism and downstream genes are induced by drought, high salinity and cold; conditions which induce ferritin too [36]. Therefore, the presence of their cognate cis-elements in the upstream sequence of *MusaFer1* may probably allow for coordinated expression of ferritin along with other stress-metabolic pathways and support plant protection. Taken together, the presence of these cognate upstream *cis*-elements coupled with oxidative stress tolerant phenotype point towards the plausible role of *MusaFer1* in regulating multiple stress pathways.

We subjected banana plants to varying levels of iron. Under high iron levels we observed induction of all *Musa* ferritins to varying folds in leaves and roots. *MusaFer1* was induced to a higher level than the other ferritins in both leaves and roots under both iron conditions. A similar observation has been made previously in *Arabidopsis* where *AtFer1* and *AtFer3* were strongly expressed under excess iron conditions [37]. *AtFer2* is unresponsive to iron levels and is specifically expressed in seeds under the effect of abscisic acid, while *AtFer1*, *AtFer2* and *AtFer3* are expressed in vegetative organs barring seeds. Phenotypically, change in root architecture in the form of increased lateral root and discoloration of the basal root part was observed. Iron stress has been known to modulate root architecture [38–40] and the death of root border cells has been purported to help protect the plant from iron toxicity; thus a similar phenomenon could be attributed to our findings where discoloration of the lower parts of roots was seen under 350 μM iron [39]. The morphological changes described in this study indicated that the iron concentrations used, probably altered the nutrient status of the medium leading to elemental imbalance and the observed phenotypic changes in the root architecture. Under iron deficiency, plants exhibited stunted roots and chlorosis of leaf tips and margins, and downregulation of all five ferritin genes in leaves. Similar changes have also been observed in *Arabidopsis thaliana* wherein, *AtFer1* and *Atfer4* were downregulated in leaves under iron deficiency [41]. A probable explanation for this is the presence of a putative IDRS *cis*-element in the promoter of *MusaFer1* which is known to repress ferritin transcription under low iron [37]. Taken together, these observations indicated iron dependent response of *Musa* ferritins.

*Agrobacterium* mediated transformation was used to generate *MusaFer1* transgenic plants. RT-qPCR was performed on these plants to estimate the overexpression levels. The quantum of overexpression did not correlate to the copy number probably due to differential sites of integration in the banana genome with varied transcription activity as the site of integration is random. Nevertheless, overexpression of *MusaFer1* increased iron content in leaf and roots of transgenic banana plants. Additionally, an analysis of the expression profile of selected iron homeostasis genes was carried out to determine the effect of overexpression of *MusaFer1* on overall iron homeostasis. It appears that ferritin overproduction may cause a ‘pseudo-deficient’ state of iron in the cell due to sequestration of available iron. Thus uptake and mobilization of iron is enhanced as evident from the induction of the *IRT* and *VIT* genes in the roots of transgenic line F3. Both genes were upregulated probably as a means to cope with the increased influx of iron and other transition elements by shunting them into the vacuole. In addition, levels of *NAS* and *YSL* were found higher than control in roots of the transgenic line. Such an induction would serve as an efficient 'push-pull mechanism' [42] to accommodate and channelize the increased iron in a safe manner. Other genes such as *FRO*, *DMAS* and *NAAT* displayed varying levels among their family members. In *Arabidopsis*, the *FRO* family consists of members with differential expression in shoot and root tissue and are generally involved in reduction of the insoluble ferric form into the soluble ferrous form of iron for uptake. The present study depicts a varied expression profile among the *FRO* homologs in the *MusaFer1* transgenic banana leaves and roots. Similar differential expression of *FRO* genes was observed in *Arabidopsis* under iron and copper treatments [43]. *NAAT* and *DMAS* gene family members together biosynthesize the phytosiderophores mugeneic acid (MA) and deoxymugeneic acid (DMA) respectively from nicotianamine in a sequential manner. Under iron deficiency, the levels of these genes are reported to increase [44]. However in the present study *MusaDMAS* in leaves was negligibly affected while in roots *MusaDMAS1* was downregulated. On the other hand *MusaNAAT1* and *MusaNAAT2* were induced and repressed respectively in both leaf and root. Overall, the differences in expression between different members of the same family may occur as a result of integrated response to the physiological perturbations imposed by excess ferritin levels as well as tissue and cell-specific expression.

In light of the observations from promoter analysis, we performed stress assays to evaluate the performance of *MusaFer1* overexpressing plants and found them more resistant to methyl viologen and high iron treatments than control plants. Methyl viologen and high iron are both potent inducers of oxidative stress [15, 45]. Our results are in line with an earlier report where transgenic tobacco overexpressing soybean ferritin was found to be tolerant to methyl viologen stress [25]. In this context, decreased levels of MDA in transgenic lines subjected to both stresses over the controls provided corroborative evidence for the protective function of *MusaFer1* in oxidative stress tolerance. This role of *MusaFer1* can provide a means to achieve increased levels of iron while still mitigating its deleterious effects.

Finally, we estimated levels of iron and zinc uptake in the transgenic lines and found that transgenic banana tissues accumulated more iron when compared with the untransformed control. Perls’ staining of petiole supported this result with more deposition of iron than the control. Similar observations previously made with soybean ferritin expressed under an endosperm- specific glutelin promoter, described increased iron and zinc content in transgenic rice [28] and by three fold in T1 seed of transgenic rice [16] compared with respective controls. Under CaMV 35S promoter it led to a 3- fold increase of iron in transgenic tobacco leaves [25]. However, relatively higher accumulation of iron at root level than in shoot of transgenic lines compared with controls could be due to immediate contact of the roots with the rhizosphere. Accumulation of iron in the initial developmental stages of one, two and three month old hardened plants was higher in root, as root iron transporters take up iron from environment into the symplast of root cells [46]. The concentration of other divalent elements such as zinc was also estimated, as *IRT* in roots is non-specific in nature and under iron deficiency secondary uptake of these elements is observed. Zinc levels were also higher compared with control, although not consistent in roots. This correlates with expression levels of *MusaFer1* in the respective transgenic line, indicating that *MusaFer1* contributed towards the increased iron content and yet maintained the homeostatic levels of iron by altered gene expression and iron flux [47].

This study has demonstrated that overexpression of the native *MusaFer1* increased iron content in leaf as well as root. As an additional benefit, tolerance towards methyl viologen and iron induced oxidative stress was also observed. This is highly relevant to the present scenario of identifying a prospective candidate for effective biofortification in banana. However, actual increase in iron content in fruit needs further investigations.

## Supporting information

S1 Fig Diagrammatic depiction of the T-DNA region of the pCAMBIA-*MusaFer1* binary vector. (TIF)

S2 Fig GUS histochemical staining. GUS histochemical staining of transformed ECS and leaf tissue. Transient overexpression seen in ECS transformed with *MusaFer1* after 5d (a), untransformed control ECS after 5d of GUS staining (b), transformed leaf tissue showing intense blue coloration after overnight GUS histochemical staining at 37⁰ C (c), untransformed control leaf tissue after overnight GUS histochemical staining at 37°C (d).
(TIF)

S3 Fig Generation of transgenic plants via somatic embryogenesis pathway. Induction of embryos on banana embryo induction medium supplemented with 5 mg/L hygromycin. Inset: magnified view of developing embryos (a), regeneration of putative transgenic shoots on banana multiplication medium supplemented with 5 mg/L hygromycin (b), multiple *in-vitro* shoots of putatively transformed *MusaFer1* lines (c), rooted *in-vitro* plantlets of putatively transformed *MusaFer1* lines (d), two month old greenhouse hardened putatively transformed *MusaFer1* lines (e).
(TIF)

S4 Fig Molecular analysis of *MusaFer1* putative transgenic lines. A 1% Agarose gel stained with ethidium bromide showing genomic DNA-PCR results of five putative transgenic lines. Lane 1 and 7 represent the untransformed control (UC) and 1kb ladder (M) respectively. Lane 2 through 6 represents the transformation events (F1, F3, F11, F17 and F22 respectively). Arrow shows 788bp amplification corresponding to *hygromycin phosphotransferase* gene residing within the T-DNA of the *MusaFer1* binary vector (a), Southern blot analysis of the five transgenic lines (F1, F3, F11, F17 and F22) and untransformed control (UC). Approximate band positions are shown using 1Kb DNA ladder (b).
(TIF)

S5 Fig ChloroP prediction of transit peptide in *MusaFer1* sequence. ChloroP result predicting presence of chloroplast transit peptide (cTP) comprising 48 amino acids.
(TIF)

S6 Fig Cleavage site prediction in transit peptide of *MusaFer1* sequence. SignalP-HMM (hidden Markov model) result predicting cleavage between 18th-19th amino acids (SEA-SQ).
(TIF)

S7 Fig *Cis*-element analysis of *MusaFer1* upstream sequence. A 1660 bp region upstream of the *MusaFer1* gene from the transcription start site (TSS) was analysed. The translation start site is boxed, TSS is indicated as +1 with an arrow mark and the nucleotides of the 5’-UTR are underlined and in bold font in the figure. The predicted TATA box is double underlined and the other motifs are highlighted as indicated. The predicted iron dependent regulatory sequence (IDRS) homologous to *ZmFer1* and *AtFer1* was identified *in-silico* in *Musa* genome sequence.
(TIF)

S1 Table Primer sequences used in the study. (DOC)

S2 Table Expression levels of *MusaFer1* in roots and leaves of transgenic lines (F1 to F22). (XLS)

S3 Table Effect of overexpression of *MusaFer1* in leaves of transgenic line F3. (XLS)

S4 Table Effect of overexpression of *MusaFer1* in roots of transgenic line F3. (XLS)

S5 Table Effect of overexpression of *MusaFer1* in roots and leaves of transgenic line F3. (XLS)
