# Supplementary figures and images for: Overexpression of native ferritin gene MusaFer1 enhances iron content and oxidative stress tolerance in transgenic banana plants
Source: PLoS One. 2017 Nov 30;12(11):e0188933. doi: 10.1371/journal.pone.0188933 (PMC5708808; doi:10.1371/journal.pone.0188933)

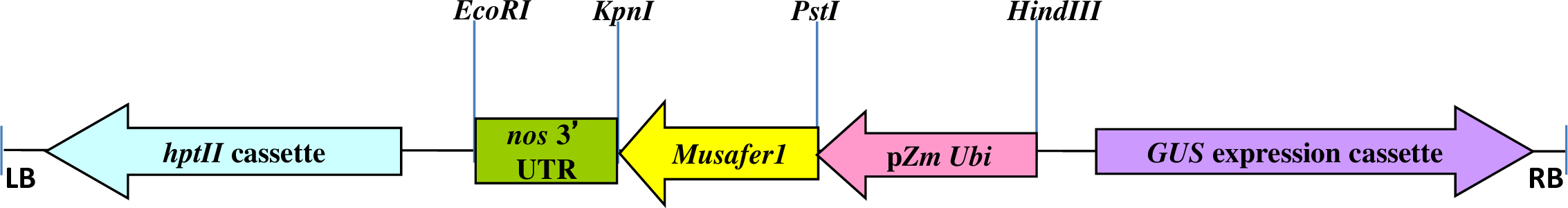

Supplement: S1 Fig — (TIF) [file pone.0188933.s001.tif]

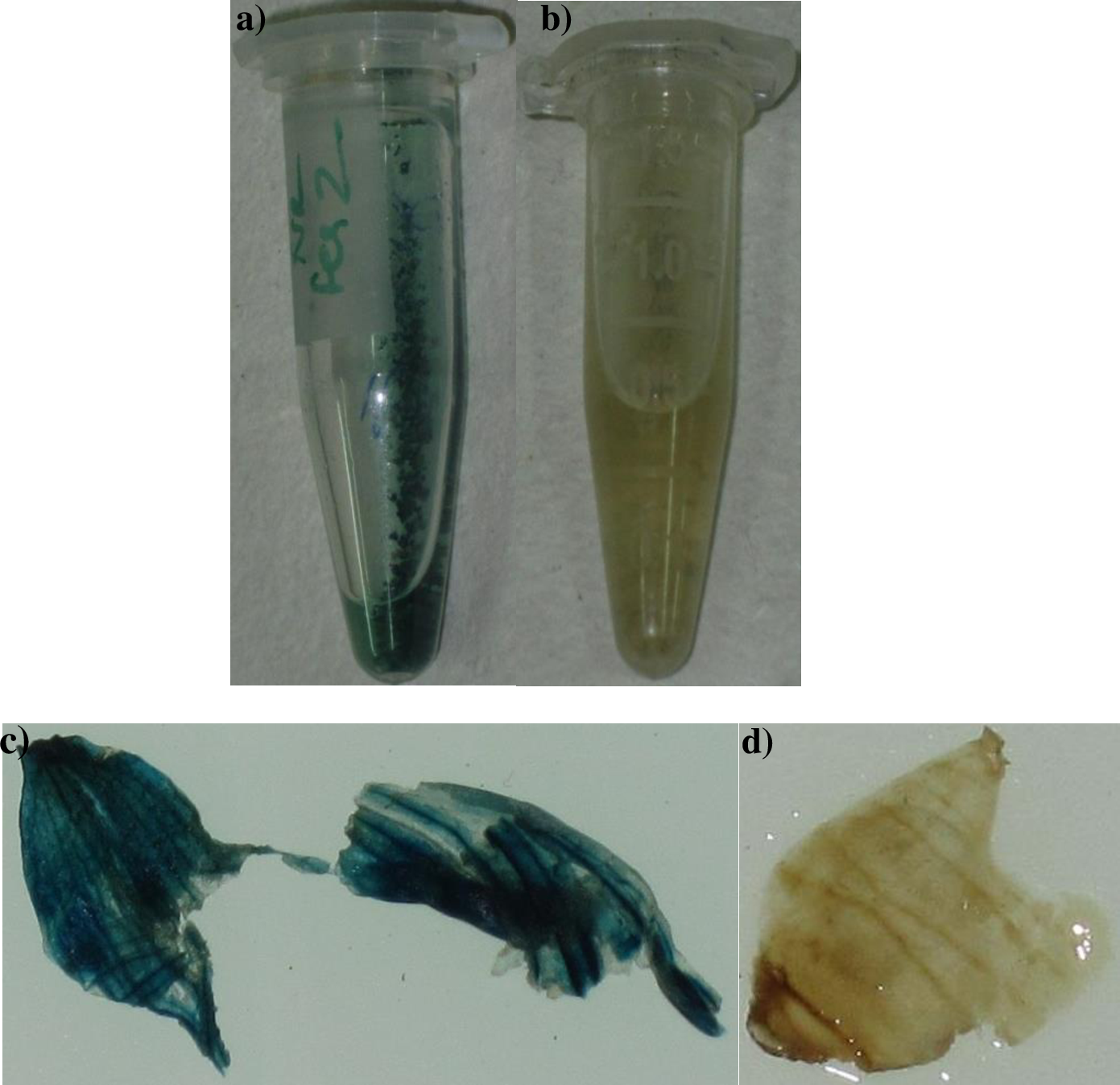

Supplement: S2 Fig — GUS histochemical staining of transformed ECS and leaf tissue. Transient overexpression seen in ECS transformed with MusaFer1 after 5d (a), untransformed control ECS after 5d of GUS staining (b), transformed leaf tissue showing intense blue coloration after overnight GUS histochemical staining at 37⁰ C (c), untransformed control leaf tissue after overnight GUS histochemical staining at 37°C (d). (TIF) [file pone.0188933.s002.tif]

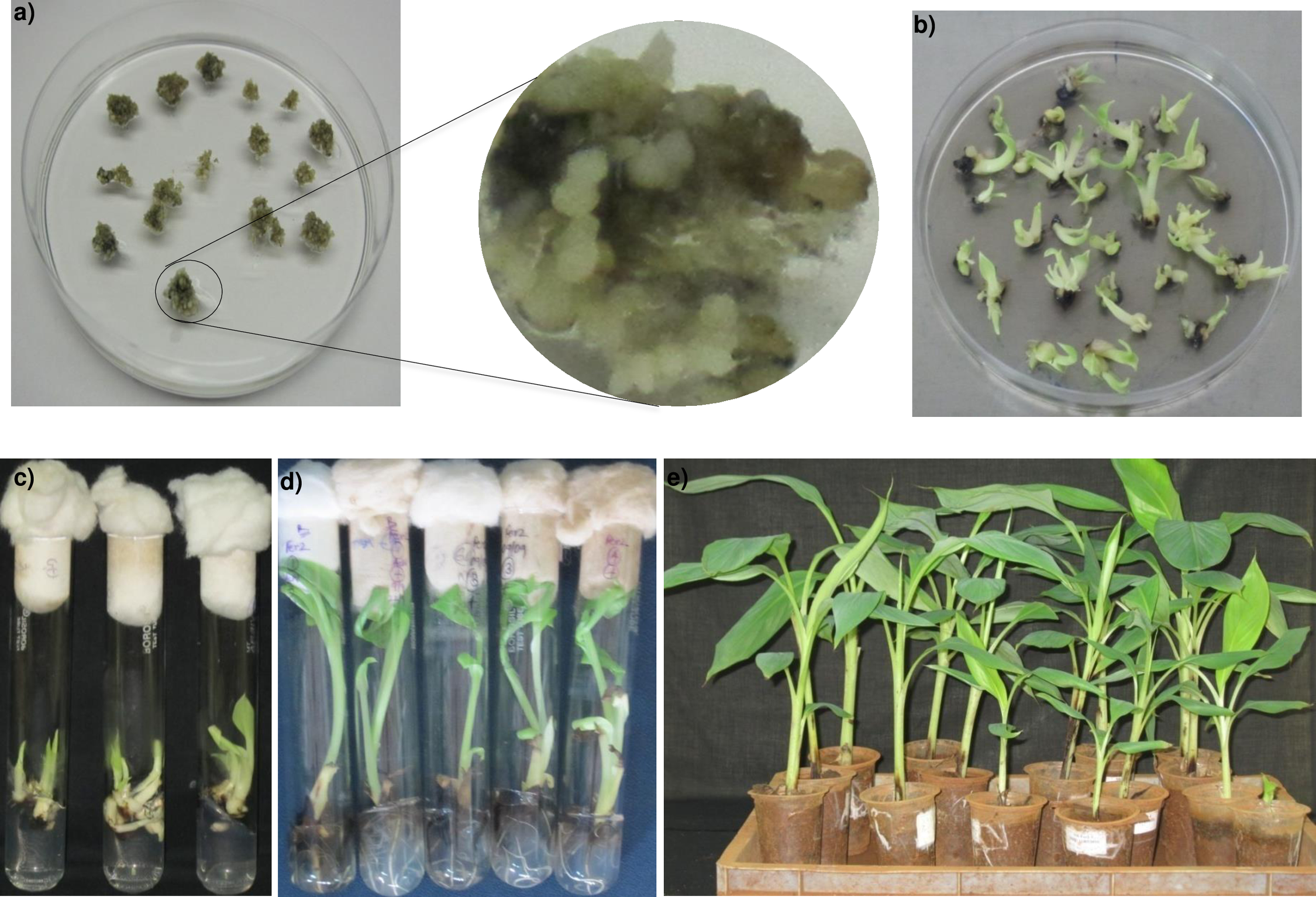

Supplement: S3 Fig — Induction of embryos on banana embryo induction medium supplemented with 5 mg/L hygromycin. Inset: magnified view of developing embryos (a), regeneration of putative transgenic shoots on banana multiplication medium supplemented with 5 mg/L hygromycin (b), multiple in-vitro shoots of putatively transformed MusaFer1 lines (c), rooted in-vitro plantlets of putatively transformed MusaFer1 lines (d), two month old greenhouse hardened putatively transformed MusaFer1 lines (e). (TIF) [file pone.0188933.s003.tif]

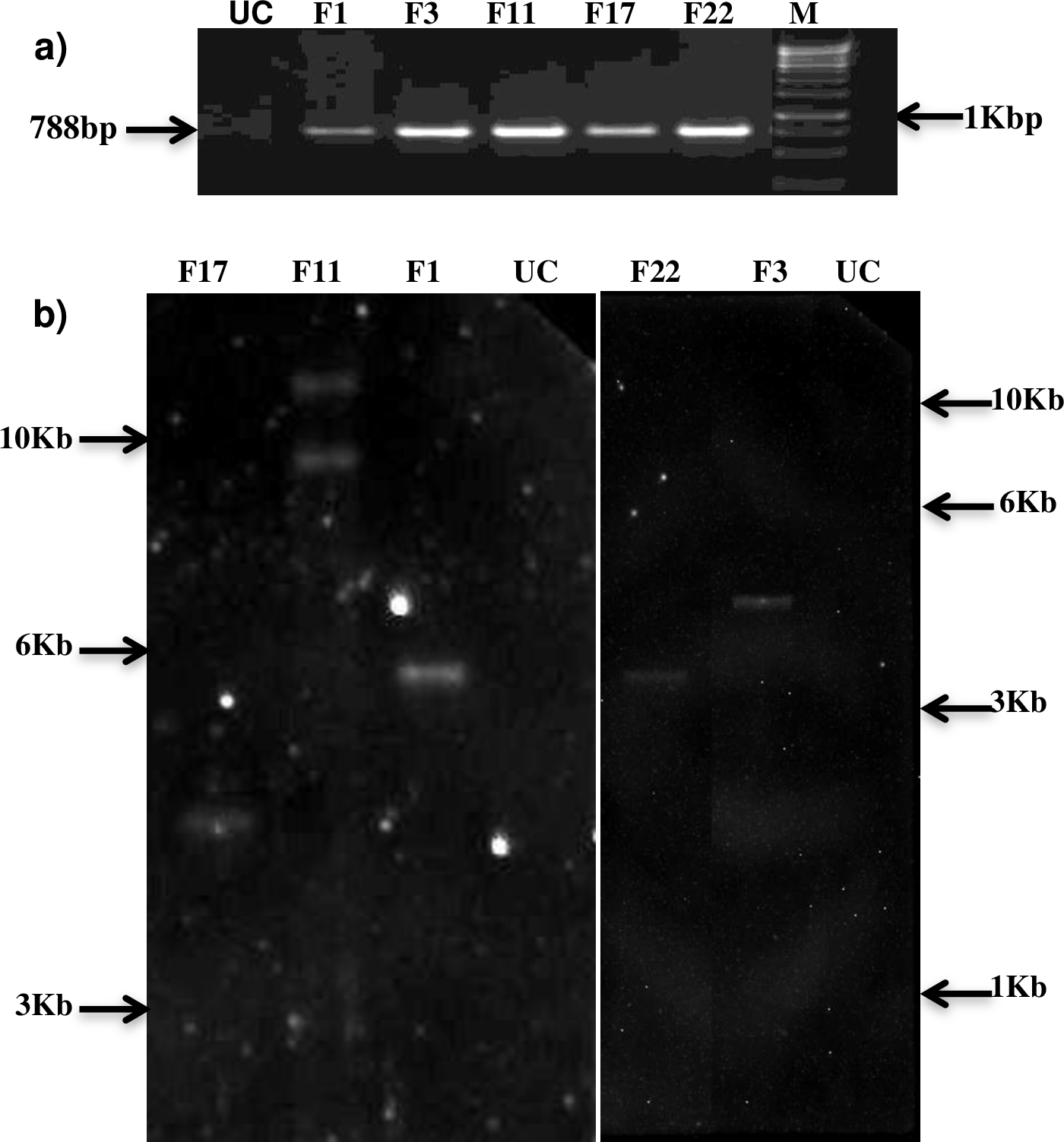

Supplement: S4 Fig — A 1% Agarose gel stained with ethidium bromide showing genomic DNA-PCR results of five putative transgenic lines. Lane 1 and 7 represent the untransformed control (UC) and 1kb ladder (M) respectively. Lane 2 through 6 represents the transformation events (F1, F3, F11, F17 and F22 respectively). Arrow shows 788bp amplification corresponding to hygromycin phosphotransferase gene residing within the T-DNA of the MusaFer1 binary vector (a), Southern blot analysis of the five transgenic lines (F1, F3, F11, F17 and F22) and untransformed control (UC). Approximate band positions are shown using 1Kb DNA ladder (b). (TIF) [file pone.0188933.s004.tif]

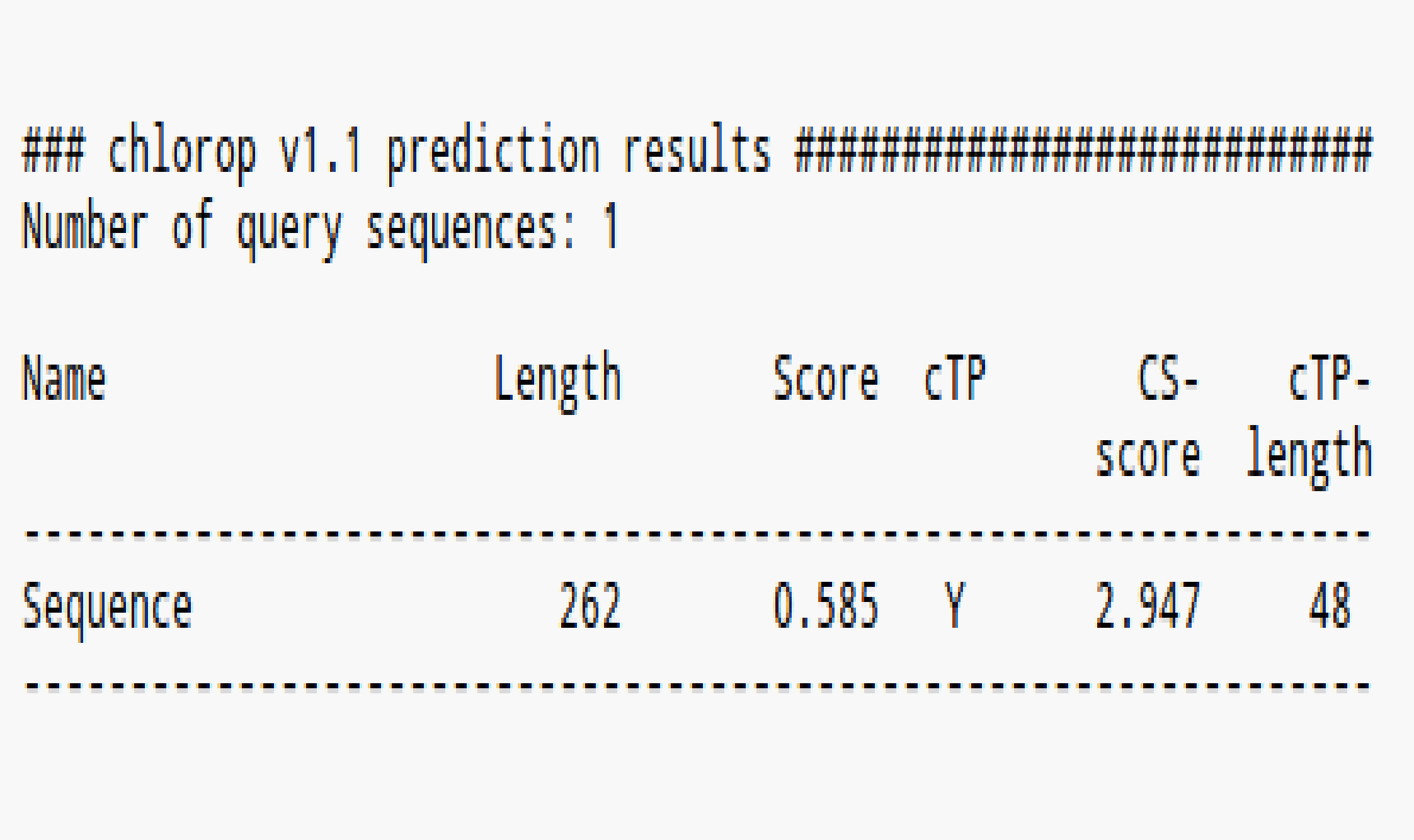

Supplement: S5 Fig — ChloroP result predicting presence of chloroplast transit peptide (cTP) comprising 48 amino acids. (TIF) [file pone.0188933.s005.tif]

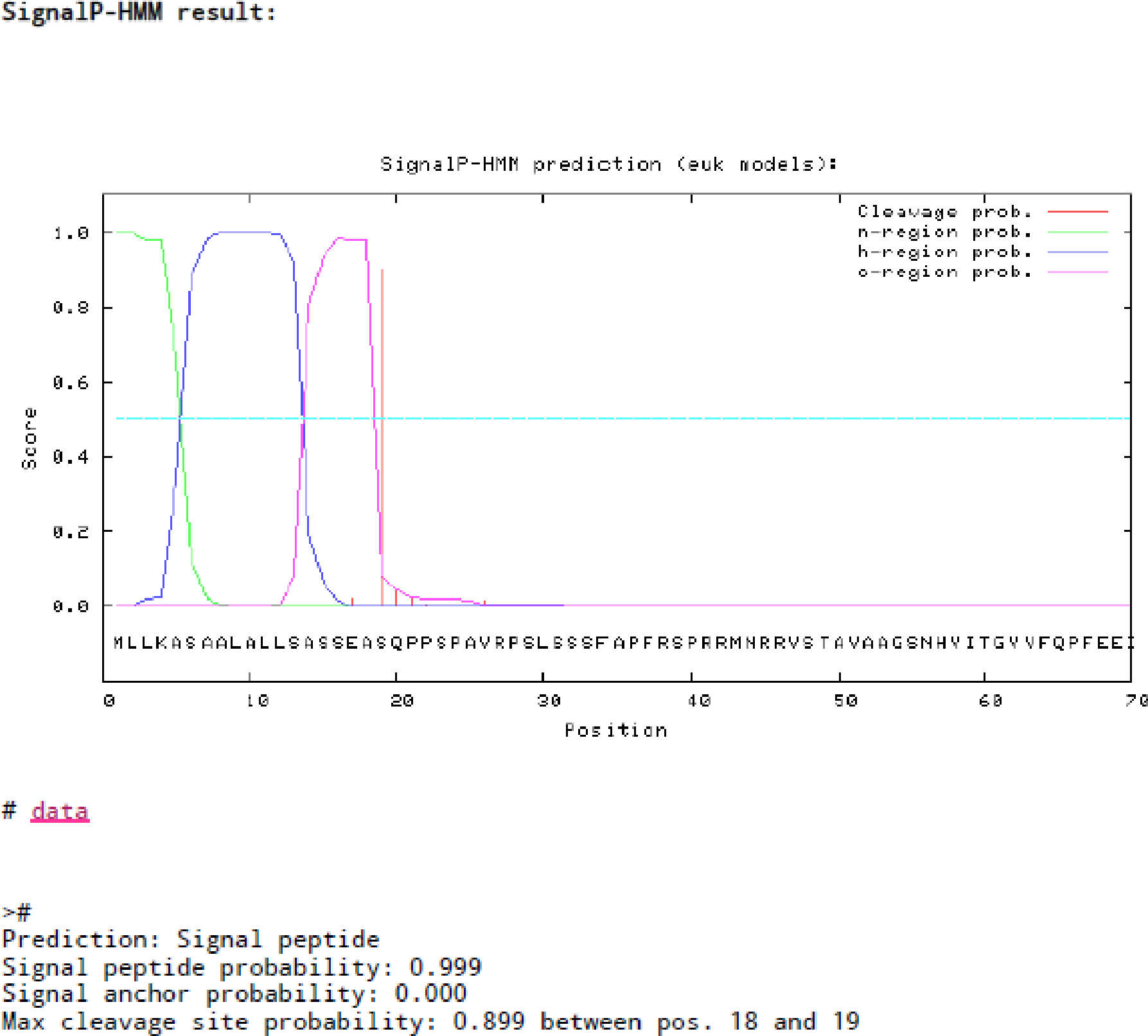

Supplement: S6 Fig — SignalP-HMM (hidden Markov model) result predicting cleavage between 18th-19th amino acids (SEA-SQ). (TIF) [file pone.0188933.s006.tif]

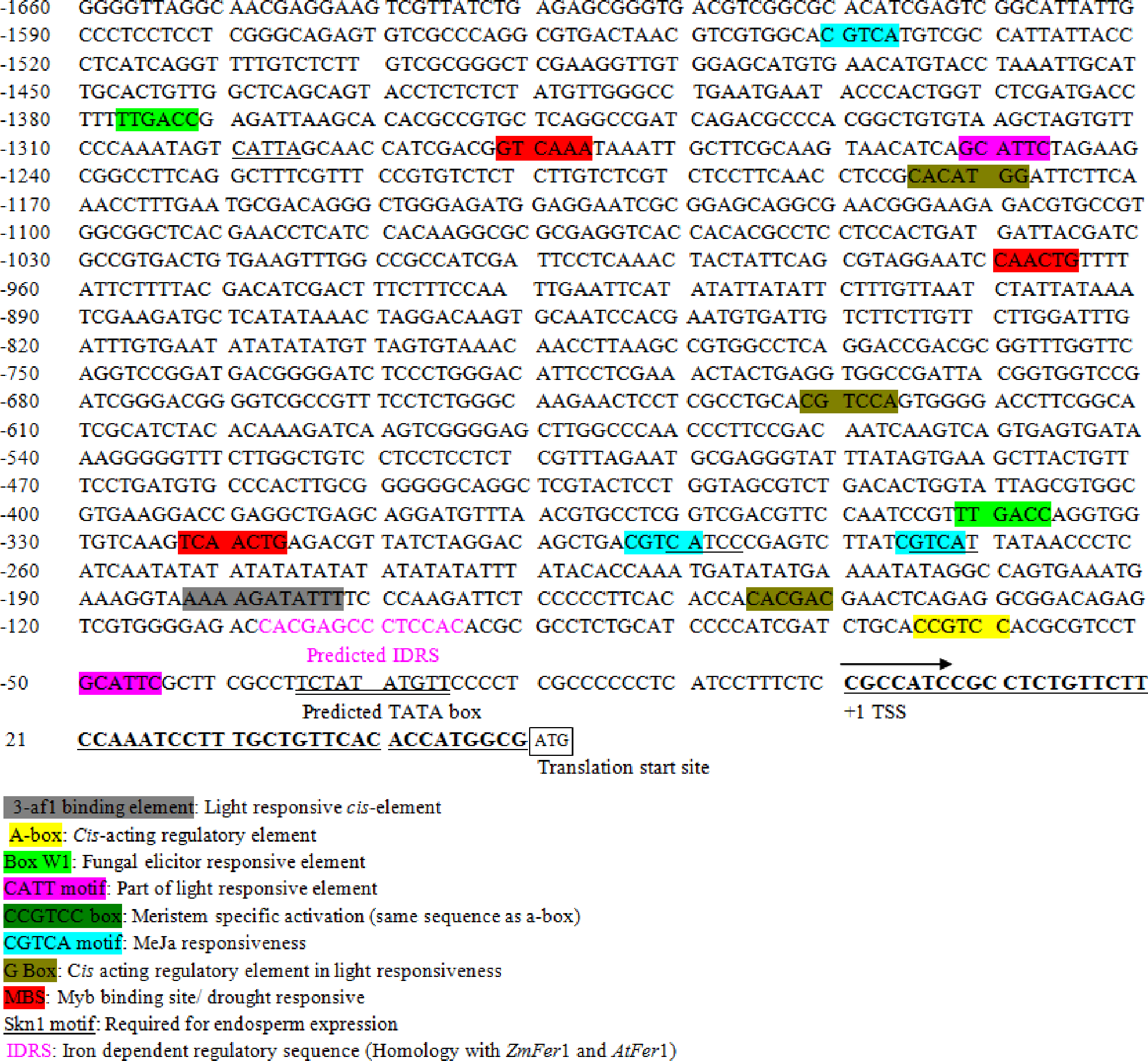

Supplement: S7 Fig — A 1660 bp region upstream of the MusaFer1 gene from the transcription start site (TSS) was analysed. The translation start site is boxed, TSS is indicated as +1 with an arrow mark and the nucleotides of the 5’-UTR are underlined and in bold font in the figure. The predicted TATA box is double underlined and the other motifs are highlighted as indicated. The predicted iron dependent regulatory sequence (IDRS) homologous to ZmFer1 and AtFer1 was identified in-silico in Musa genome sequence. (TIF) [file pone.0188933.s007.tif]
