# Supplementary material for: Overexpression of native ferritin gene MusaFer1 enhances iron content and oxidative stress tolerance in transgenic banana plants
Source: PLoS One. 2017 Nov 30;12(11):e0188933. doi: 10.1371/journal.pone.0188933 (PMC5708808; doi:10.1371/journal.pone.0188933)
Supplement: S1 Table — (DOC) [file pone.0188933.s008.doc]

Supplementary table 1

Amplification and Real time primers used in the study

| Number | Primers | Primer sequence 5’🡪3’ |
| --- | --- | --- |
| Amplification forward (Fw) and reverse (Rv) primer for *MusaFer1* and *Hygromycin* CDS | | |
| 1 | *MusaFer1* CDS  AFw | CTGCAGATGCTTCTCAAGGCCTCCGCCGCTCTC |
| 2 | *MusaFer1* CDS  ARv | GGTACCCTATGCCTGATGCAACTTTCCTTCATCCAGCAGCA |
| 3 | *Hygromycin* Fw | GTCCTGCGGGTAAATAGCTG |
| 4 | *Hygromycin* Rv | ATT TGTGTACGCCCGACAGT |
| Real time *Musa* amplification forward (Fw) and reverse (Rv) primers | | |
| 5 | *Efα* Fw | CCGATTGTGCTGTCCTCATT |
| 6 | *Efα* Rv | TTGGCACGAAAGGAATCTTCT |
| 7 | *IRT1*Fw | ACG GGA TGA AGA TGA GAG GC |
| 8 | *IRT1*Rv | CGT AAGCCCAGAGTTGAAGC |
| 9 | *IRT2* FW | CTTCAGGCTGAGTACGGGAT |
| 10 | *IRT2* Rv | TGCAGCTTAGGACCCATGAA |
| 11 | *VIT1* FW | GGGAGCAAGAGGAGATCGTT |
| 12 | *VIT1* Rv | CACGACGTAGGAGAGAGCAA |
| 13 | *VIT2* FW | AGGTCGGCGAGTTACTGTC |
| 14 | *VIT2* Rv | GATGAAGACGTAGGGCAGGA |
| 15 | *FRO21* FW | GGCATCCTCCAGCGTTACTA |
| 16 | *FRO21* RV | TCCGCATTGTAGAACCAGGA |
| 17 | *FRO22* FW | CGATGAGAAGGAACGAACCC |
| 18 | *FRO22* Rv | GTGGCTGTTGAAGTGGAAGA |
| 19 | *FRO23* FW | CTCTGCTCGTTGGCCATTG |
| 20 | *FRO23* Rv | CACACACGAAGACACCCACT |
| 21 | *FRO24* FW | CCTCTTTTGTCGCATTCCTCG |
| 22 | *FRO24* Rv | AACCAGAAACCGGGCGAA |
| 23 | *FRO25* FW | TAGTCTGGCTGCATGGAAGA |
| 24 | *FRO25* Rv | AGAGGTCCCCAAGTAATGCC |
| 25 | *FRO26* FW | TCACCATGCTCGATCTCCTC |
| 26 | *FRO26* Rv | GGAATGCGACGAAAGAGGAC |
| 27 | *NAAT1* Fw | TCACAACAGATCCTGCAACC |
| 28 | *NAAT1* Rv | GGATGCCTTCCAAGTGAGAC |
| 29 | *NAAT2* Fw | ACCATCAGTGTCTTGAGGCA |
| 30 | *NAAT2* Rv | CTTGAATCCCACAGCACACC |
| 31 | *DMAS1* Fw | CCAAGGGGATTCAAGTGTGT |
| 32 | *DMAS1* Rv | TCCAGCATCCTCTTCTCGTT |
| 33 | *DMAS2* Fw | TTGGGTTCGACTGGGACTC |
| 34 | *DMAS2* Rv | CTTCTGCAACTCTTCGGCTG |
| *35* | *NAS 2* Fw | TCCAAGGAAGTGAACGAGCT |
| *36* | *NAS 2* Rv | TGTACTCCAAAAGGCTGAGC |
| 37 | *NAS 1* Fw | ATCAGCCACGACGACAAGG |
| 38 | *NAS 1* Rv | AGGGCCTCATCACCGCTG |
| *39* | *NAS 3* Fw | CTCTGCCTCTGACCTCCATC |
| *40* | *NAS 3* Rv | TCGATCACCCGGACCTTCT |
| 41 | *NAS 4* Fw | GACGACAAGATCCGCGTG |
| 42 | *NAS 4* Rv | AGT ACTTGCAGGGCCTCAC |
| 43 | *YSL6* Fw | GCAGTGCCTTTCTACATTGGA |
| 44 | *YSL6* Rv | CAGATTGGGGGATCAATCCTA |
| 45 | *YSL8* Fw | CATGGCCATACCGTTCTACC |
| 46 | *YSL8* Rv | GGCGTCTACCTTGTCGTTGT |
| *47* | *MusaFer1* Fw | CCGAGTTTAATCACGCCGAG |
| *48* | *MusaFer1* Rv | CGATCAAAGTGCCAAACCCC |
| 49 | *MusaFer*5 Fw | GCAATGGAATTGGCTTTGTC |
| 50 | *MusaFer*5 Rv | TGGAGAAGCATCTGATCGAA |
| 51 | *MusaFer4* Fw | ATCCTGAGAAAGGCGATGC |
| 52 | *MusaFer4* Rv | AAATGCCAGACTCCATGTCC |
| 53 | *MusaFer2* Fw | TGGCGTTGTGTCTTGAGAAG |
| 54 | *MusaFer2* Rv | TCTCCCTGAAGGAGCATCTG |
| 55 | *MusaFer3* Fw | GGAGATTGCCTTGTCTCTCG |
| 56 | *MusaFer3* Rv | TCCTTCACCAAAAAGCACCT |
| 57 | *YSL12* Fw | CCATCGACATGTGTGTAGGC |
| 58 | *YSL12* Rv | CTCATTTCCACACACGGATG |
